# African dryland antelope trade‐off behaviours in response to heat extremes

**DOI:** 10.1002/ece3.11455

**Published:** 2024-06-06

**Authors:** Paul Berry, Melanie Dammhahn, Morgan Hauptfleisch, Robert Hering, Jakob Jansen, Anna Kraus, Niels Blaum

**Affiliations:** ^1^ Plant Ecology and Nature Conservation, Institute of Biochemistry and Biology University of Potsdam Potsdam Germany; ^2^ Behavioural Biology, Institute for Neuro‐ and Behavioural Biology (INVB) University of Münster Münster Germany; ^3^ Research Directorate Namibia Nature Foundation Windhoek Namibia; ^4^ Unit for Environmental Sciences and Management North West University Potchefstroom Nort West Province South Africa; ^5^ Biodiversity Research Centre Namibia University of Science and Technology Windhoek Namibia; ^6^ Ecology/Macroecology, Institute of Biochemsitry and Biology University of Potsdam Potsdam Germany

**Keywords:** accelerometer data, behavioural responses, climate change, mammals, thermoregulation

## Abstract

Climate change is predicted to narrow the prescriptive zone of dryland species, potentially leading to behavioural modifications with fitness consequences. This study explores the behavioural responses of three widespread African antelope species—springbok, kudu and eland—to extreme heat in a dryland savanna. We classified the behaviour of 29 individuals during the hot, dry season on the basis of accelerometer data using supervised machine learning and analysed the impact of afternoon heat on behaviour‐specific time allocation and overall dynamic body acceleration (ODBA), a proxy for energy expenditure, along with compensatory changes over the 24‐hour cycle. Extreme afternoon heat reduced feeding time in all three antelope species, increased ruminating and resting time, while only minimally affecting walking time. With rising heat, all three species reduced ODBA on feeding, while eland reduced and kudu increased ODBA on walking. Diel responses in behaviour differed between species, but were generally characterised by daytime reductions in feeding and increases in ruminating or resting on hot days compared to cool days. While antelope compensated for heat‐driven behavioural change over the 24‐hour cycle in some cases, significant differences persisted in others, including reduced feeding and increased rumination and resting. The impact of heat on antelope behaviour reveals trade‐offs between feeding and thermoregulation, as well as between feeding and rumination, the latter suggesting a strategy to enhance nutrient uptake through increased digestive efficiency, while the walking response suggests narrow constraints between cost and necessity. Our findings suggest that heat influences both behaviour‐specific time allocation and energy expenditure. Altered diel behaviour patterns and incomplete compensation over the 24‐hour cycle point to fitness consequences. The need to prioritise thermoregulation over feeding is likely to narrow the prescriptive zone of these dryland antelope.

## Graphical abstract

This study examines the impact of extreme heat on the behaviour of three African antelope species in a dryland savanna. It finds that extreme afternoon heat reduces feeding time, increases ruminating and resting time, but minimally affects walking time across all species, while affecting energy expenditure on active behaviours. While some compensations for heat‐driven behavioural changes occur over the 24‐hour cycle, significant differences persist, suggesting potential fitness consequences.

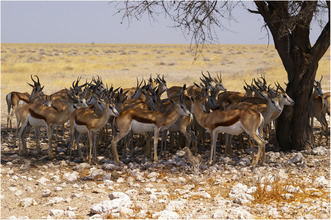

## 1 INTRODUCTION

Animals inhabiting drylands are subject to harsh environmental conditions, such as high temperatures, intense solar radiation, sparse shade, limited precipitation and a scarcity of surface water (Fuller et al., 2021; Mendelsohn et al., 2022). The imperative to avoid hyperthermia in dryland regions has driven the evolution of diverse heat stress mitigation strategies, including morphological, physiological and behavioural adaptations (Blank & Li, 2022). Over time, mammals indigenous to these areas have become superbly adapted (Fuller et al., 2014). However, the current trajectory of rapid climate change, coupled with land degradation (Scholes, 2009) and movement barriers (Hering et al., 2022), presents unparalleled challenges. In some dryland regions, average temperatures have risen by more than 1°C within just three decades (Turner et al., 2022). Additionally, warmer temperatures have a drying effect by increasing evaporative losses (Cook et al., 2018). Furthermore, projected increases in temperature and the occurrence of droughts and heatwaves will exacerbate these already extreme conditions (Naumann et al., 2018; Perkins‐Kirkpatrick & Lewis, 2020; Wei et al., 2019). This intensified warming and drying may impact the prescriptive zone of dryland mammals—the ambient temperature range conducive to maintaining a relatively constant core body temperature (Mitchell et al., 2018). Beyond mere thermoregulatory challenges, alterations in behaviour under extreme heat may have far‐reaching consequences for fitness and survival (Fuller et al., 2021).

The most immediate response of animals to rapid environmental change is typically behavioural (Hetem et al., 2014; Wong & Candolin, 2015). Under heat stress, mammals exhibit altered behaviours such as increased resting (Terrien, 2011), seeking shade (Cain et al., 2006), orienting their bodies relative to the sun (Hetem et al., 2011; Hofmeyr & Louw, 1987) and reducing movement and foraging activity (Brivio et al., 2019). Some species may shift their activity from the hottest times of the day to cooler periods, transitioning to crepuscular and nocturnal activity (Du Toit & Yetman, 2005; Scheibe et al., 2009). While certain species effectively adjust their daily activity without compromising foraging time (Semenzato et al., 2021), others may struggle to fully compensate (Trondrud et al., 2023). This may potentially lead to decreased food intake (Fuller et al., 2016; Shively et al., 2019), thereby limiting the energy and nutrients available for homeostasis, growth and reproduction (Schneider, 2004).

Many studies on behavioural heat response focus on single species. However, the variety of behavioural responses described above highlights the value of comparative studies on sympatric species exposed to the same conditions in a common study area. Such studies can help identify species‐specific as well as general challenges in adapting to future extreme heat and dryness and potentially inform targeted conservation strategies for vulnerable species.

We focus on three sympatric African antelope species—springbok (*Antidorcas marsupialis*), greater kudu (*Tragelaphus strepsiceros*) and eland (*Tragelaphus oryx*)—living in a semi‐arid savanna. They are valuable to the eco‐tourism and hunting sectors, contributing to the growing importance of wildlife‐based land utilisation in savannas (Naidoo et al., 2016). The three species differ in various aspects such as body size, mobility, habitat and foraging preferences and play pivotal roles in savannas, influencing vegetation, other herbivores, predators, soil dynamics and susceptibility to fire (Gordon et al., 2019). A recent study (Berry et al., 2023) examined how antelope respond to extreme heat in terms of their activity levels, but the underlying behavioural trade‐offs involved remained unclear. Since such trade‐offs are key to understanding the capacity of species to meet the demands of thermoregulation in a warming climate, our aim in this study was to compare the behavioural responses of African antelope to extreme dryland heat.

Behaviour has traditionally been studied through direct observation, but recent advancements in biologging now enable around‐the‐clock, non‐invasive and bias‐free data collection on a large scale (Chakravarty et al., 2019; Glass et al., 2020; Nathan et al., 2012, 2022; Studd et al., 2019). Consequently, behaviour classification on the basis of accelerometer (ACC) data has successfully been applied to mammals (Chakravarty et al., 2020; Chimienti et al., 2021; Kirchner et al., 2023), birds (Patterson et al., 2019; Yeap et al., 2021), reptiles (Fossette et al., 2010; Whitney et al., 2021) and fish (Beltramino et al., 2019; Clarke et al., 2021). Here we classify antelope behaviour based on ACC data and relate this to rising temperature. Based on recent findings on activity levels (Berry et al., 2023), we expected that (1) antelope would reduce the time spent on active behaviours, such as feeding and walking, with rising heat. In addition, we hypothesised that (2) heat responses encompass changes not only in the time allocated to, but also the energy expended per unit time on, different behaviours. Furthermore, we expected (3) antelope on hot days to partially compensate for any daytime reductions in active behaviours by shifting them to cooler times.

## 2 METHODS

### 2.1 Study area

**FIGURE 1:**
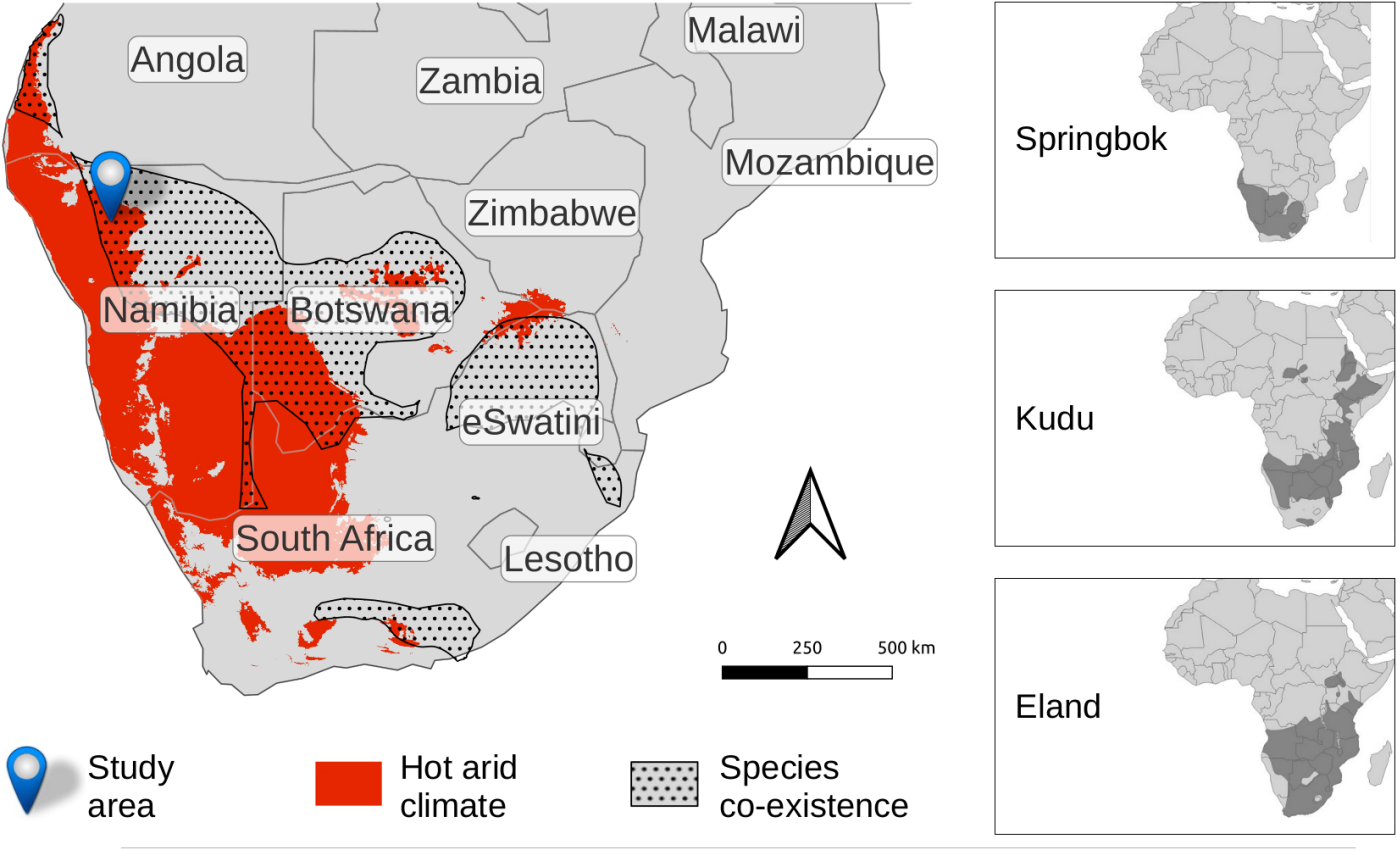
The behavioural responses of three antelope species to extreme heat were studied in the Etosha area of northern Namibia (blue pin), a hot arid climate zone in which the three species co‐exist. The hot arid climate zone (Köppen‐Geiger climate classification) is indicated in red (Beck et al., 2023), with the overlapping habitat of the species shown by dots. Insets on the right display the entire distribution of each species (Jetz et al., 2012).

Our primary study site was Etosha Heights private reserve, centred on 19.2° S, 15.2° E, and bordering on Etosha National Park in northern Namibia. For practical reasons however, direct observations of animal behaviour for the labelling of ACC data were done at Sophienhof private reserve, centred on 20.1° S 16.1° E, approx. 130 km south‐east of Etosha Heights. Given this proximity, the two sites are ecologically similar to each other. Notably, this area is among the hottest and driest in which the three study species occur simultaneously (Figure 1). The average maximum temperature of the area is 32°C–34°C, the sites are located between the 300 and 350 mm mean annual rainfall isohyets while the annual potential evaporation amounts to 2400–2500 mm, the area is elevated approx. 1000 m above sea level, soils comprise predominantly calcisols and leptosols, and the vegetation is classified as Karstveld tree and shrub savanna (Mendelsohn et al., 2022). Land use at both sites focuses on eco‐tourism. The reserves are fenced, have distributed artificial water points and offer a diversity of wildlife typical to the region.

### 2.2 Study species

Springbok (*A. marsupialis*), kudu (*T. strepsiceros*) and eland (*T. oryx*) are widespread across southern Africa (Skinner & Chimimba, 2005). Body size varies widely among the three species, with female body mass of springbok, kudu and eland approximating 40 kg, 150 kg and 300 kg, respectively (Skinner & Chimimba, 2005). Adapted to arid conditions, all three species are largely independent of drinking water if water content in food is adequate, though they will drink if surface water is available (Nagy & Knight, 1994; Skinner & Chimimba, 2005). Springbok are classed as intermediate feeders (Robbins et al., 1995) and prefer open grassland habitat. Kudu are classed as concentrate selectors (Hofmann, 1989) and prefer savanna woodland habitat for its cover and availability of browse (Skinner & Chimimba, 2005). Like springbok, eland are classed as intermediate feeders (Hofmann, 1989), showing great versatility in diet, and occurring in a wide range of habitats throughout southern Africa, except where mean annual precipitation is less than 300 mm (Skinner & Chimimba, 2005).

### 2.3 Recording ACC data

Eleven springbok, nine kudu and nine eland were fitted with GPS collars equipped with ACC modules (e‐obs GmbH, Grünwald, Germany) on the Etosha Heights reserve. On the Sophienhof reserve, an additional three springbok, one kudu and one eland were fitted with the same collars in order to label accelerometer data with behaviour by direct observation. All study animals were adult females with the exception of one eland bull at Etosha Heights. The collars were fitted during chemical immobilisation by wildlife veterinarians registered in Namibia and the study was approved by the Namibian National Commission on Research, Science & Technology (certificate number RCIV00032018, with authorisation numbers: 20190602, 20190808, and AN202101048).

For animals at Etosha Heights, collars were configured to record tri‐axial ACC data for 3.3 s every 5 min at a sampling frequency of 33.3 Hz, resulting in 110 ACC values per axis for each recording. To facilitate labelling of ACC data by direct observation on Sophienhof, collars there were configured to record ACC data for 6.6 s every 30 s, also at a sampling frequency of 33.3 Hz. Each 6.6 second‐long recording was subsequently divided into two for further processing.

### 2.4 Labelling ACC data

To label the ACC data with behaviour for supervised machine learning (Nathan et al., 2012), direct observations were done at Sophienhof during daylight hours in October and November 2021. Animals were located by a radio signal emitted by the collar and cautiously approached by two observers in a farm vehicle to which they were habituated, typically to a distance of 50–100 m. The collared individual was filmed from the vehicle during ACC recording while keeping disturbance to a minimum.

The timestamps of the accelerometer data and video material were synchronised. The event‐logging software *Boris* (Friard & Gamba, 2016) was used to label accelerometer recordings with behavioural states of the collared animal. Twelve types of behaviour were discerned: browsing, drinking, foraging, grazing, grooming, low‐activity, ruminating, running, salt‐licking, sleeping, trotting and walking. Foraging—slow walking with the apparent intent of searching for food—was distinguished from feeding—food intake by cropping and chewing while standing. Ruminating was defined as pro‐longed chewing and occasional regurgitation not flanked before and after by feeding. Low‐activity included both standing and lying. Running in eland and sleeping in kudu and eland were observed too seldom for these behaviours to be included in the further analysis. A detailed ethogram is given in Table S1 in the Appendix S1.

Labelling ACC data with behaviour on only a few individuals (three springbok, one kudu and one eland, see Section 2.3) means they could have been unusual in their gait or behaviour patterns. However, a subsequent analysis on the proportion of time spent on different behaviours inferred on the individuals of each species at Etosha Heights was both plausible as well as consistent among individuals.

### 2.5 Preparing a behaviour classifier

The steps we took to prepare an automated classifier of behaviour on the basis of ACC data included: ACC data visualisation to verify correct labelling, calculation and subsequent selection of features used to train the classification model, model training and performance testing. We used the R package *rabc* (Yu & Klaassen, 2021) for all of these tasks.

#### 2.5.1 ACC data visualisation

To visualise the ACC data, we used the *plot_acc* function of the *rabc* package, which sorts ACC data by behaviour and plots the ACC data so that any anomalies in the patterns, which may point to incorrect labelling, become apparent (Yu & Klaassen, 2021).

#### 2.5.2 Feature calculation

Acceleration was measured in units ranging from 0 to 4095, corresponding to −4 G to 4 G (G denoting the Earth's gravitational acceleration, 9.81 m/s^2^). These raw values were centred around zero by subtracting 2048 units (a value of 2048 corresponding to no acceleration).

To account for varying sensor orientations in the x‐ and y‐axes (surge and sway) due to differing collar fitment, mean values for the x and y axes were compared between the training set from Sophienhof and the inference dataset of each of the Etosha Heights study animals. The orientations of the x and y axes were reversed on the inference dataset, if applicable.

To account for collar rotation around the neck, the magnitude of acceleration in the yz‐plane $a_{\mathrm{yz}}$ was calculated by taking the square root of the sum of squares of the acceleration components of the y‐ and z‐axes, $a_{y}$ and $a_{z}$:

$$a_{\mathrm{yz}}=\sqrt{a_{y}^{2}+a_{z}^{2}}$$

For each 3.3 s continuous recording, features were calculated to form the input of the machine learning model (Hastie et al., 2009). The features were calculated separately for the x‐axis as well as for the yz‐plane, except for ODBA (Overall Dynamic Body Acceleration), which was calculated using all three axes. The following time‐domain features were calculated: mean, variance, standard deviation, maximum, minimum, range, and ODBA, a proxy of energy expenditure (Wilson et al., 2019). The following frequency‐domain features were calculated: main frequency, main amplitude, and frequency entropy (a measure of the unpredictability of the signal). The calculations were done using the respective functions contained in the *rabc* package. The length of the running window for the ODBA calculation was set to 22 samples (1/5th of a recording, 0.67 s), and the sampling frequency was set to 33.3 Hz.

#### 2.5.3 Feature selection

We used the *select_features* function in the *rabc* package to select the five most predictive features. The function removes redundant features based on the absolute values of the pair‐wise correlation coefficients between features (Yu & Klaassen, 2021). The threshold correlation coefficient was set to the default of 0.9, so that the resulting features had correlations below this threshold value. The selected features and their contribution to classification accuracy were as follows, for springbok: x‐variance: 0.650, x‐mean: 0.148, yz‐entropy: 0.032, x‐freqmain: 0.018, yz‐min: 0.009; for kudu: x‐max: 0.697, x‐freqamp: 0.128, x‐freqmain: 0.029, yz‐variance: 0.009, yz‐entropy: 0.007; and for eland: yz‐variance: 0.748, x‐min: 0.088, x‐freqmain: 0.020; x‐entropy: 0.008, x‐mean: 0.007.

#### 2.5.4 Model training

The machine learning model for behaviour classification was based on a gradient boosting decision tree algorithm implemented by the XGBoost library (Chen & Guestrin, 2016). We used the *rabc* package to train the model using the five selected features. The labelled dataset comprised 3952 observations for springbok (1188 observations for ID 8316, 1366 observations for ID 8318 and 1398 observations for ID 8320), 2406 observations for kudu (ID 8319) and 2876 observations for eland (ID 7297). The *train_model* function automatically deals with machine learning model hyperparameter tuning, model training and evaluating model performance with a test dataset. As per default setting, the labelled data were randomly split into 75% training data and 25% test data. The hyperparameters were left at their default values: nrounds = 10, max_depth = 6, eta = 0.3, gamma = 0, colsample_bytree = 1, min_child_weight = 1, subsample = 1 (Chen & Guestrin, 2016; Yu & Klaassen, 2021).

#### 2.5.5 Model testing

**FIGURE 2:**
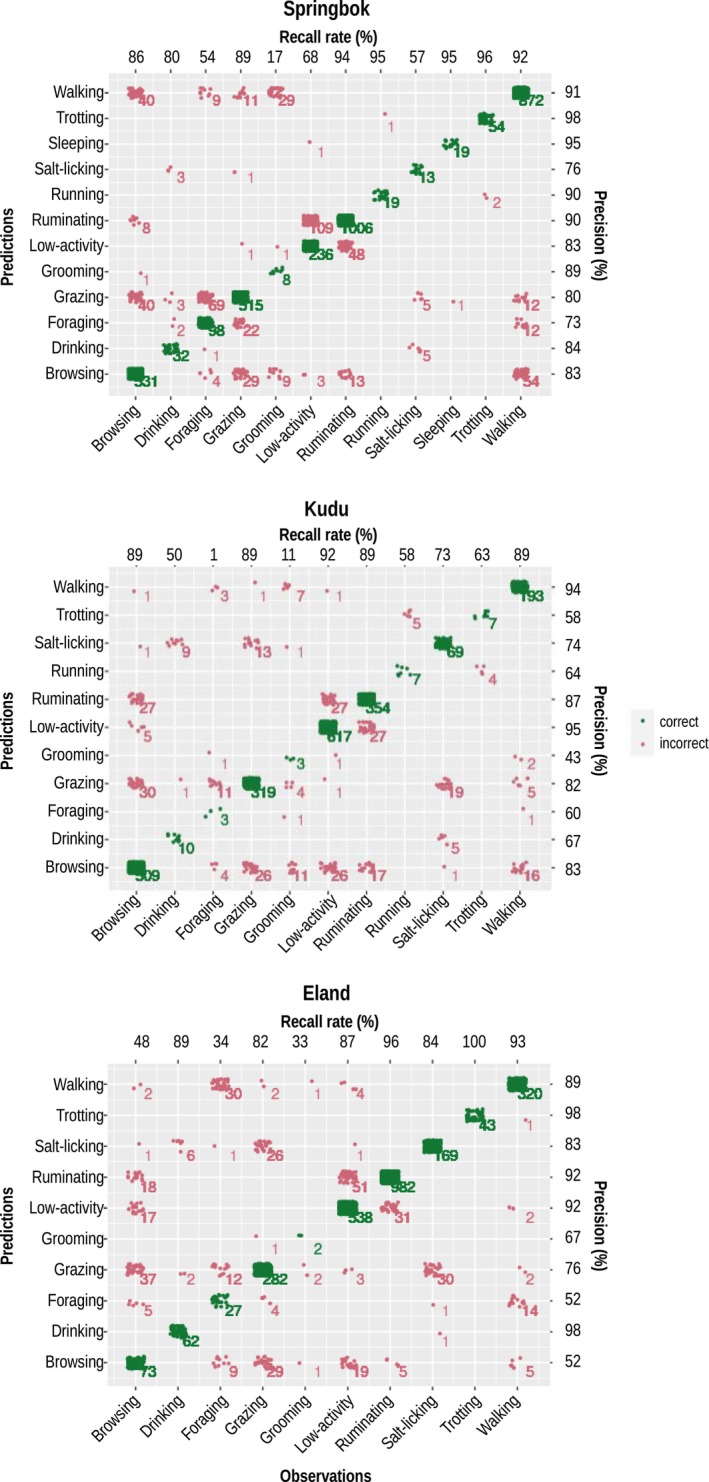
Confusion matrix plots showing the performances of the behaviour classifiers trained on springbok (top), kudu (middle) and eland (bottom). Green dots on the main diagonal show correct predictions, red dots off the diagonal show incorrect predictions with accompanying coloured numbers indicating the number of predictions for each category. Recall rates are given in the top row and precision in the right column of each panel.

The performance of the behavioural classification model was assessed using the *plot_confusion_matrix* function of the *rabc* package. The function employs cross‐validation, randomly partitioning the dataset into five parts and training the model five times, each time using a different part as the validation set and the remaining four parts as the training set. The classification models yielded an accuracy of 84%, 95% CI [82%, 87%] for springbok, 87%, 95% CI [85%, 90%] for kudu and 86%, 95% CI [83%, 89%] for eland. The confusion matrix plots show the observed behaviours in columns and the predicted behaviours in rows, so that the correct predictions fall on the main diagonal of the plot, and the incorrect predictions off the main diagonal (Figure 2). Precision and recall rates are also given.

### 2.6 Prediction of behaviour

With the trained classifier, we used the *rabc predict* function to infer behaviour from unlabelled ACC data recorded on Etosha Heights study animals. To focus on heat extremes, we selected October–December as the hottest months of the year (Berry et al., 2023). For springbok, ACC data for the October–December period were available for 2019 and 2020, and for kudu and eland for 2020. A total of 245,549 behaviour predictions were done for springbok, 311,954 for kudu and 263,742 for eland.

### 2.7 Analysis of behaviour

#### 2.7.1 Behavioural response to afternoon heat

We analysed data for the afternoons (12:00–17:59), which are the hottest part of the day (Berry et al., 2023). We calculated blackglobe temperature—a commonly used measure of heat stress—as a function of air temperature, solar radiation and relative humidity (Hajizadeh et al., 2017), which were measured at a weather station at the study site. Furthermore, we accounted for the effects of time of day, time of year and individual. We thus quantified overall dynamic body acceleration (ODBA), a proxy for energy expenditure (Wilson et al., 2019), for each combination of degree Celsius (blackglobe), hour of the day (across several days), month of the year and individual. For the further analysis, we calculated the mean ODBA values if they were based on at least 30 recordings (equating to 100 s of measurement).

For our analysis, the 12 different behaviours were consolidated into five main categories. Foraging (searching for food while slowly walking) also occurred relatively seldom (approx. 4%, 1% and 5%, for springbok, kudu and eland, respectively) and was therefore combined with walking. Sleeping was directly observed only in springbok. Together with low activity, it was categorised as resting. Drinking, salt‐licking, grooming, trotting and running in combination occurred only ~2% of the time and were therefore excluded in the further analysis. Given our focus on the major behavioural categories, the potential for lower predictive classification performance for minority categories (Kaur et al., 2020) was considered to have minimal impact on our study.

For each of five distinct behaviours (browsing, grazing, walking, ruminating and resting), we used a generalised additive mixed model (GAMM) to estimate the proportion of time allocated to that behaviour, resulting in five GAMMs. Each GAMM estimated the proportion of time allocated to the respective behaviour as a function of species, and its interactions with blackglobe temperature, hour of the afternoon, and month of the year, while treating individuals as random effects. By including the month and hour variables in the models, we accounted for possible temporal structures in the data. For the analyses of behaviour‐specific energy expenditure, we modelled mean ODBA for each of the five behaviours as a function of the same predictors as above, resulting in an additional five GAMMs. Concurvity levels were below 0.8. The significance of each parameter in explaining the variation in the response variables was assessed by *p*‐value with *α* = .05. All statistical analyses were done in the R programming language (version 4.2.3; R Core Team, 2023), and we used the R package *mgcv* (version 1.8–42) for GAMM analyses.

#### 2.7.2 The effect of temperature on behaviour over the 24‐hour cycle

To illustrate how extreme heat affects behaviour over the 24‐hour cycle, we compared the proportion of time spent on the five different behaviours for each species between the 10 hottest and 10 “coolest” days within hottest months of the year (October–December). For each species dataset, we identified these extreme days according to daily maximum air temperature. For the 10 “coolest” days, this averaged 30.2°C (range 26.0°C–32.4°C), corresponding to a blackglobe temperature of 45.0°C (38.8°C–49.8°C) in the case of springbok for which data were available for 2019 and 2020, and 32.6°C (range 29.5°C–34.3°C), corresponding to a blackglobe temperature of 48.5°C (41.3°C–53.2°C), in the case of kudu and eland for which data were available for 2020. For the 10 hottest days, daily maximum air temperatures averaged 39.9°C (range 39.5°C–40.6°C), corresponding to a blackglobe temperature of 58.7°C (57.2°C–60.6°C), in the case of springbok, and 39.6°C (range 39.1°C–40.6°C), corresponding to 57.8°C (56.5°C–60.1°C), in the case of kudu and eland. These extreme days were spread relatively evenly across the months studied. For each of the three species and five behaviours, we used a GAMM to model the proportion of time spent on the specific behaviour as a function of hour of the day, as well as its interaction with the hot day / cool day factor, and treated individuals as random effects. We regarded the diel patterns in activity to differ between hot and cool days if the interaction terms were significant, and we regarded the mean activity over the 24‐hour cycle to differ between hot and cool days if the intercepts differed significantly.

## 3 RESULTS

**FIGURE 3:**
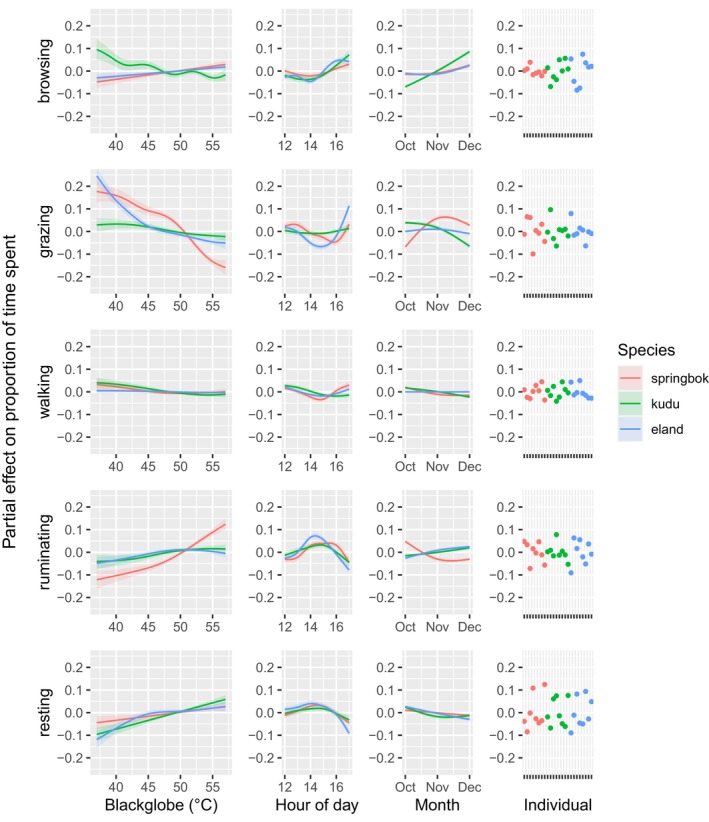
Partial effects of blackglobe temperature, hour of the afternoon, month (October–December) and individual on time spent browsing, grazing, walking, ruminating and resting in springbok, kudu and eland (shown in blue, green and red, respectively), as estimated by Generalised Additive Models.

Extreme afternoon heat affected the time allocated to behaviours, with differences across the three species. Figure 3 shows the partial effects of blackglobe temperature, hour of the afternoon, month and individual on the proportion of time spent on activities during the hottest times of the day.

Rising afternoon temperature was associated with an overall decrease in time spent feeding across all three species. The mixed feeders springbok and eland substantially decreased grazing and slightly increased browsing, while kudu, which are predominantly browsers, slightly decreased browsing. Walking was affected only minimally in all three species. Ruminating increased with rising temperature for all three species, substantially so in the case of springbok. Likewise, resting generally increased with rising temperature. The smooth terms involving temperature were generally significant (Table 1), though effect sizes varied among behaviours and species (Figure 3). Complete model summaries including parametric coefficients, as well as smooth terms for all predictor variables, are given in Table S2 in Appendix S1.

**TABLE 1:** Generalised additive mixed models (GAMMs) were used to estimate the effect of various predictor variables on the proportion of time spent on five different behaviours for springbok, kudu and eland.

Behaviour	Term	Edf	Ref. df	*F*‐value	*p*‐Value
Browsing	s(blackglobe):springbok	0.930	9.000	1.677	.0001***
	s(blackglobe):kudu	5.660	9.000	5.627	.0000***
	s(blackglobe):eland	0.888	9.000	1.435	.0027**
Grazing	s(blackglobe):springbok	4.985	9.000	44.146	.0000***
	s(blackglobe):kudu	2.139	9.000	2.718	.0001***
	s(blackglobe):eland	4.673	9.000	33.730	.0000***
Walking	s(blackglobe):springbok	2.255	9.000	1.054	.0068**
	s(blackglobe):kudu	2.293	9.000	3.641	.0000***
	s(blackglobe):eland	0.811	9.000	0.192	.1323
Ruminating	s(blackglobe):springbok	3.063	9.000	20.945	.0000***
	s(blackglobe):kudu	2.195	9.000	2.780	.0005***
	s(blackglobe):eland	2.441	9.000	2.507	.0003***
Resting	s(blackglobe):springbok	0.902	9.000	1.282	.0012**
	s(blackglobe):kudu	0.976	9.000	13.505	.0000***
	s(blackglobe):eland	3.542	9.000	9.214	.0000***

*Note*: Here only the smooth term of the effect of temperature on each behaviour is reported; complete model summaries including parametric coefficients and smooth terms involving hour, month and individual, are given in the Appendix S1. **p < .01, ***p < .001.

Abbreviations: edf, estimated degrees of freedom; Ref.df, reference degrees of freedom.

**FIGURE 4:**
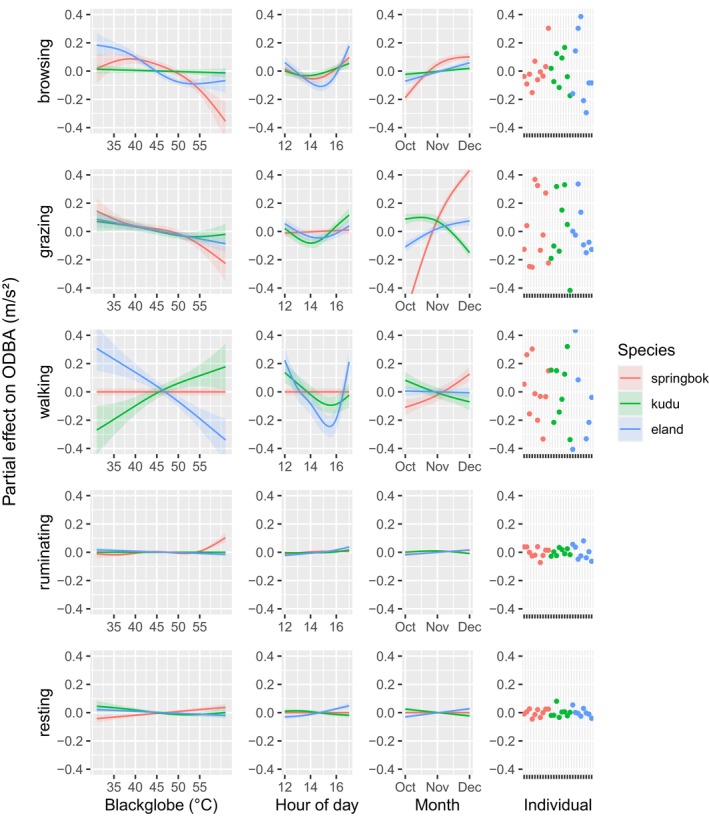
Partial effects of blackglobe temperature, hour of the afternoon, month (October–December) and the individual on energy expenditure per unit time overall dynamic body acceleration (ODBA) in the case of (from top to bottom) browsing, grazing, walking, ruminating and resting for springbok, kudu and eland (shown in blue, green and red, respectively).

In addition to changing time allocation, rising afternoon heat in many cases also changed the behaviour‐specific ODBA, a proxy for energy expenditure (Figure 4). ODBA was reduced in browsing in the case of the mixed feeders springbok and eland and ODBA in grazing was reduced in all three species as temperatures rose. Eland substantially reduced ODBA, while kudu increased ODBA, associated with walking as temperatures rose (Figure 4). Summaries of the effects of temperature on the ODBA of different behaviours are given in Table 2. Detailed model summary including all predictors is given in Table S3 in the Appendix S1.

**TABLE 2:** Generalised additive mixed models (GAMMs) were used to estimate the effect of various predictor variables on the overall dynamic body acceleration (ODBA) spent on five different behaviours for springbok, kudu and eland.

Component	Term	Edf	Ref. df	*F*‐value	*p*‐Value
Browsing	s(temp):springbok	2.849	4.000	16.866	.0000***
	s(temp):kudu	0.427	4.000	0.189	.1808
	s(temp):eland	2.198	4.000	18.935	.0000***
Grazing	s(temp):springbok	2.506	4.000	8.859	.0000***
	s(temp):kudu	1.425	4.000	1.624	.0111*
	s(temp):eland	0.900	4.000	2.451	.0014**
Walking	s(temp):springbok	0.000	4.000	0.000	.4841
	s(temp):kudu	1.386	4.000	3.745	.0001***
	s(temp):eland	1.305	4.000	8.156	.0000***
Ruminating	s(temp):springbok	3.729	4.000	11.349	.0000***
	s(temp):kudu	0.000	4.000	0.000	.4123
	s(temp):eland	0.870	4.000	1.920	.0053**
Resting	s(temp):springbok	0.913	4.000	2.824	.0007***
	s(temp):kudu	1.594	4.000	2.334	.0025**
	s(temp):eland	0.775	4.000	0.893	.0334*

*Note*: Here only the smooth term of the effect of temperature on each behaviour is reported; complete model summaries including parametric coefficients and smooth terms involving hour, month and individual, are given in the Appendix S1. *p < .05, **p < .01, ***p < .001.

Abbreviations: edf, estimated degrees of freedom; Ref.df, reference degrees of freedom.

**FIGURE 5:**
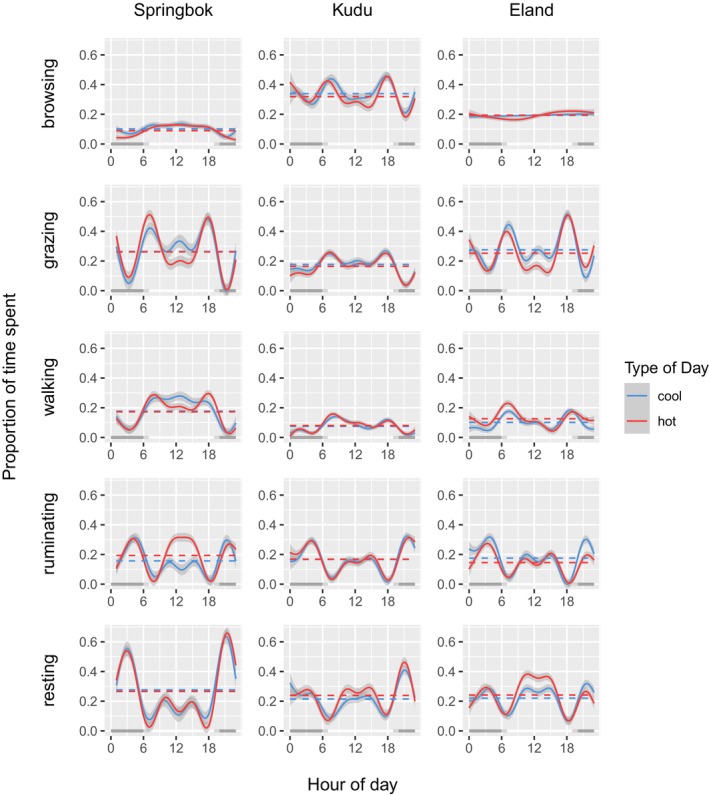
A comparison of the proportion of time spent on various activities between hot (red) and cool (blue) days over the 24‐hour cycle for springbok (left), kudu (centre) and eland (right), with 24‐hour means shown by horizontal dotted lines. For the cool days, maximum blackglobe temperature averaged 45.0°C (38.8°C–49.8°C) in the case of springbok, and 48.5°C (41.3°C–53.2°C) in the case of kudu and eland. For the hot days, maximum blackglobe temperature averaged 58.7°C (57.2°C–60.6°C), in the case of springbok, and 57.8°C (56.5°C–60.1°C), in the case of kudu and eland.

During the hottest 3 months of the year (October–December), diel patterns in times spent on behaviours generally differed significantly between hot days and cool days, except in kudu in the case of grazing, walking and ruminating (Table 3). Daytime reductions in active behaviours were apparent (browsing in kudu, grazing in springbok and eland, walking in springbok), while daytime increases in behaviours of low‐activity (ruminating in springbok, resting in kudu and eland) were apparent on hot days compared to cool days (Figure 5). Notably, springbok spent more time grazing in the early mornings, while spending less time grazing and walking but more time ruminating during the middle of the day. Kudu spent less time browsing and more time resting during daytime and eland spent less time grazing and more time resting during daytime (Figure 5).

**TABLE 3:** Differences between hot and cool days in diel patterns and means over the 24‐hour cycle as shown in Figure 5 are summarised here for five different behaviours.

Behaviour	Springbok			Kudu			Eland		
	Diel pattern	24‐h means/% difference		Diel pattern	24‐h means/% difference		Diel pattern	24‐h means/% difference	
Browsing	*	*	−12	*	*	−6	*		
Grazing	*				*	−7	*	*	−7
Walking	*						*	*	20
Ruminating	*	*	21				*	*	−17
Resting	*			*	*	11	*	*	11

*Note*: Percentage differences of 24‐h means on hot days relative to cool days are given for significant cases.

* Statistical significance: *p* < .05.

Changes in behaviour‐specific time allocation in response to daytime heat were in some cases compensated for over the 24‐hour cycle. However, significant differences in averages over the 24‐hour cycle remained in the following cases: reduced browsing and increased rumination in springbok, reduced feeding and increased resting in kudu, and reduced grazing and ruminating but increased walking and resting in eland (Table 3). Complete model summaries are given in Table S4 in the Appendix S1.

## 4 DISCUSSION

Studying the effect of heat extremes on antelope behaviour in a dryland savanna, we explored responses both during the heat of the day and over the 24‐hour cycle. This approach enabled us to identify daytime responses, potential shifts of specific behaviour to night‐time as well as the degree of compensation over the 24‐hour cycle. Our findings show that the studied antelope species generally reduce feeding time and increase resting as well as rumination time in response to daytime heat, but that walking time is relatively unaffected. Furthermore, not only does heat impact the way individuals allocate their time for certain behaviours but it also appears to affect their energy expenditure per unit time on those behaviours, assuming that overall dynamic body acceleration (ODBA) serves as a reliable indicator (Wilson et al., 2019). Altered diel behaviour patterns under heat and incomplete compensation over the 24 h cycle suggest potential fitness consequences.

### 4.1 Feeding responses correspond to habitat preferences

As expected, our results show that high temperatures lead to a significant decrease in feeding time during the hottest period of the day. Springbok and eland decreased grazing time and slightly increased browsing time with rising heat load. This corresponds to these species moving from sun‐exposed open areas to seeking the shade of woody vegetation during hot afternoons (Hofmeyr & Louw, 1987; Skinner & Chimimba, 2005). Rises in afternoon temperatures least affected feeding time in kudu, reflecting that kudu occupy woody habitat that shields them from the thermal stress of solar radiation (Owen‐Smith, 1998). Hence, the trade‐off between feeding and thermoregulation reflects the species' respective habitat preferences. In addition, springbok and eland reduced their ODBA spent feeding, perhaps indicating an energy‐saving strategy in response to rising temperatures.

Our observations of reduced foraging activity at elevated temperatures align with other observations in various ungulate species. In a seasonal comparison, Kirk's dik‐dik antelope (*Madoqua kirkii*) foraging time was limited by high midday temperatures (Manser & Brotherton, 1995). In kudu, active and foraging times appeared to be restricted when daily maximum air temperature exceeded 30°C in the dry season (Owen‐Smith, 1998). Likewise, steenbok (*Raphicerus campestris*), impala (*Aepyceros melampus*), kudu and giraffe (*Giraffa camelopardalis*) decreased feeding time at elevated temperatures (Du Toit & Yetman, 2005), and Arabian oryx (*Oryx leucoryx*) spent less than two hours feeding on days with maximum temperatures above 40°C, compared with 4.8 hours on cooler days (Seddon & Ismail, 2002). These studies were done by direct observation and therefore gave important insights to behavioural changes mainly during daytime. However, our approach additionally enabled us to identify potential shifts of specific behaviour to night‐time as well as the degree of compensation over the 24‐hour cycle.

We observed that on hot days antelope compensated for reduced afternoon foraging by shifting activity to cooler times of the day. On hot days, springbok increased their feeding in the early morning, possibly in anticipation of the afternoon heat, during which they reduced feeding activity. Such anticipatory behaviour has also been proposed for ibex (*Capra ibex*) (Aublet et al., 2009). However, when considering overall time allocation over the 24‐hour cycle, a trade‐off in feeding and other behaviours persisted, with variations among species. Generally, total feeding time decreased on hot days, traded‐off against increased resting in kudu and eland, increased ruminating in springbok and increased walking in eland. These changes in overall time allocation to different behaviours in response to heat paint a more nuanced picture than emerged in the study on activity levels (Berry et al., 2023) and may indicate species‐specific strategies to cope with heat extremes.

### 4.2 Increased rumination as an adaptive strategy

While rising afternoon temperature resulted in a predicted decrease in feeding and increase in resting time, it surprisingly also led to an increase in rumination time. Though negative correlations between times spent feeding and ruminating have been observed in dairy cows (Dado & Allen, 1994; Schirmann et al., 2012), they have been attributed to the mutual exclusivity of the two behaviours (Schirmann et al., 2012). Notably however, our study suggests that temperature drives the trade‐off between feeding and ruminating, with effect sizes varying across species. The pronounced increase in afternoon rumination time observed in springbok in response to heat extends to an overall elevation in rumination time on hot days. This stands in contrast to dairy cows, where heat stress led to a reduction in total rumination time, with the most significant decrease occurring during the heat of the day (Abeni & Galli, 2017). Like dairy cows, eland reduce overall rumination time on hot days, however primarily through reduced night‐time rumination. Similarly, Soriani et al. (2013) reported a negative correlation between rumination time and daily maximum heat stress in dairy cows, but they recorded a shift towards increased rumination during night‐time. One possible explanation for the substantial increase in springbok rumination on hot days is that they may be compensating for reduced food intake by enhancing digestive efficiency. This is supported by studies in dairy cattle, where rumination is associated with a reduction in feed particle size, facilitating microbial digestion in the rumen and aiding the movement of small particles through the digestive tract (Beauchemin, 2018).

### 4.3 Narrow constraints on walking

Contrary to our prediction, the time allocated to walking was largely unaffected by rising temperature during the afternoon period. However, slight decreases in walking time during daytime were observed in springbok, accompanied by a shift towards sunset hours on hot days. In eland, temperature minimally affected afternoon walking time, but ODBA suggests the energy expended on walking was reduced. Over the 24‐hour cycle, eland spent more time walking during early morning and night on hot days compared to cool days, resulting in a slight overall increase in walking time. An increase in nocturnal walking in eland, but not springbok, may reflect the comparatively low predation risk for eland (Veldhuis et al., 2020). The relatively small effect of heat on walking time may suggest narrow constraints bounded on the one hand by necessity and on the other by energetic, water balance and thermoregulatory costs. Movement serves to access resources such as food and water and visiting waterholes has been shown to increase travel to differing degrees between ungulate species (Boyers et al., 2021; Owen‐Smith & Goodall, 2014). Species also differ in their frequency of drinking, some only drinking every couple of days (Cain et al., 2012), also depending on the context (Brooks et al., 2008; Owen‐Smith et al., 2020). Perhaps the decision of whether or not to travel to a waterhole may be influenced more by how long ago the last waterhole visit was than by temperature.

### 4.4 Persistent behavioural change may affect fitness

Despite the pronounced impact of temperature on behaviour during the hottest times of the day, a comparison of behaviour time budgets over the 24‐hour cycle between hot and cool days suggests animals can largely compensate for reductions during the heat of the day by shifting active behaviours to cooler times. The varied response of the studied antelope species to high temperatures indicates differing capacities and strategies to cope with heat stress. Kudu show the least behavioural response presumably because their preference for woody habitat provides them with both food and shade. In contrast, springbok and eland, both more partial to open habitat than kudu, need to trade off feeding under sun exposure with thermoregulation demands. Though a seasonal shift in diet that may also influence habitat selection, this was accounted by including month of the year (October to December) in the models. Contrary to our expectations, springbok over the 24‐hour cycle compensated daytime changes in activity to a greater extent than eland. These findings raise the question of what changes in behaviour mean. Do kudu show little response to heat because they are less affected or more constrained by their habitat and food preference? Do springbok show greater compensation over the 24‐hour cycle than eland because they are under greater pressure to do so or because they are better adapted to coping with heat? Moreover, potential synergistic effects of time and energy allocation need to be considered. Additionally, factors such as water availability and predator presence likely influence the trade‐offs between thermoregulation, nutrition, and predation risk (Fuller et al., 2016). This complexity highlights the challenge of predicting how animals will cope with elevated temperatures.

### 4.5 Conclusion

Climate change will narrow the prescriptive zone of dryland mammals (Fuller et al., 2021), and our results suggest that this could also impact the antelope populations studied. The behavioural responses the animals exhibited will most likely reduce food acquisition. This effect may be multiplied by a drier climate impacting plant biomass and hence food availability. Such reduced energy intake could in turn elevate the lower bound of the prescriptive zone, potentially making animals susceptible to radiative heat loss during the relative cold of the night (Berry et al., 2023; Fuller et al., 2021). In addition, altered behaviour could have cascading effects on the ecosystem. For instance, changes in feeding behaviour and selectivity of feeding plants may affect the vegetation (Staver et al., 2021) and shifts in feeding to cooler times of day may interact with predation risk (Valeix et al., 2009). As the study area is located in one of the hottest and driest parts of the antelopes' common distribution, and as such is characterised by a lack of food and water, the populations there will likely be among the most challenged by further rises in temperature. It remains to be clarified to what extent the rising temperatures to which these populations are subjected impact fitness and at what point management measures may be needed. Measures such as the installation of additional artificial waterpoints should be carefully managed, since they are likely to lead to piospheres, spatially concentrated forms of land degradation (Hess et al., 2020; Thrash & Derry, 1999). This highlights the need for further investigation into the potential long‐term ecological effects of rising temperatures in the light of continued change.

## AUTHOR CONTRIBUTIONS

**Paul Berry:** Conceptualization (equal); data curation (equal); formal analysis (lead); investigation (equal); methodology (equal); project administration (equal); validation (lead); visualization (lead); writing – original draft (lead); writing – review and editing (equal). **Melanie Dammhahn:** Conceptualization (supporting); formal analysis (supporting); methodology (equal); supervision (supporting); validation (supporting); visualization (supporting); writing – original draft (supporting); writing – review and editing (equal). **Morgan Hauptfleisch:** Data curation (equal); funding acquisition (supporting); resources (supporting); writing – review and editing (equal). **Robert Hering:** Data curation (equal); investigation (supporting); visualization (supporting); writing – review and editing (equal). **Jakob Jansen:** Data curation (equal); investigation (supporting); writing – review and editing (equal). **Anna Kraus:** Data curation (equal); investigation (supporting); writing – review and editing (equal). **Niels Blaum:** Conceptualization (equal); data curation (equal); formal analysis (supporting); funding acquisition (lead); investigation (equal); methodology (equal); project administration (equal); resources (lead); supervision (lead); validation (supporting); visualization (supporting); writing – original draft (supporting); writing – review and editing (equal).

## FUNDING INFORMATION

This work formed part of the ORYCS project in the SPACES II programme which was supported by the German Federal Ministry of Education and Research (Grant No. FKZ01LL1804A). Paul Berry was funded by the SPACES II.2 CaBuDe scholarship programme of the German Academic Exchange Service (Programme No. 57531823).

## CONFLICT OF INTEREST STATEMENT

None.

## Supporting information


Appendix S1

## Data Availability

The data are available on the Movebank online platform: https://www.movebank.org, Movebank ID 904829042.
