# Supplementary material for: African dryland antelope trade‐off behaviours in response to heat extremes
Source: Ecol Evol. 2024 Jun 6;14(6):e11455. doi: 10.1002/ece3.11455 (PMC11157150; doi:10.1002/ece3.11455)
Supplement: Supplementary file 1 [file ECE3-14-e11455-s001.odt]

Supporting Material

# Ethogram

Table S1: Ethogram of 12 behaviours showing the number of labelled ACC recordings for the three study species.

| Behaviour | Description | Springbok | Kudu | Eland |
| --- | --- | --- | --- | --- |
| Browsing | Cropping and chewing shrub vegetation with neck held along or above the body axis while standing. | 620 | 573 | 153 |
| Drinking | Drinking from a waterhole at ground level with the neck tilted downward while standing. | 40 | 20 | 70 |
| Foraging | Slow walking with the apparent intent of searching for food, head held near ground level or close to vegetation. | 181 | 22 | 79 |
| Grazing | Feeding at ground level while standing. Cropping and chewing grasses or vegetation at ground level while the neck is tilted downward. | 579 | 359 | 344 |
| Grooming | Nibbling, licking, or scratching with horns, on shoulders, flanks and hindquarters while standing, neck bent towards rump. | 47 | 27 | 6 |
| Low-activity | Standing or lying, but not ruminating. Usually observed as resting in midday heat or sometimes as vigilance in between other behaviours. Neck tilted along or above the axis of the body. | 349 | 673 | 616 |
| Ruminating | Pro-longed chewing and occasional regurgitation while standing or lying and not flanked before and after by feeding; neck tilted along or above own body axis. | 1067 | 398 | 1018 |
| Running | High-speed locomotion with instances in which all feet are off the ground simultaneously (cantering or galloping). | 20 | 12 | 2 |
| Salt-licking | Licking salt stones while standing; neck tilted downward. | 23 | 94 | 201 |
| Sleeping | Lying with neck bent towards hind legs, head resting on the ground. | 20 | 0 | 0 |
| Trotting | Medium-speed locomotion with bouncy steps, faster than walking but slower than running; neck tilted upward. | 56 | 11 | 43 |
| Walking | Locomotion at the speed of human walking, with at most two feet off the ground at once, sometimes while chewing; neck in varying positions. | 950 | 217 | 344 |

# Analysis of afternoon behaviour-specific time allocation

Table S2: Generalised Additive Model summaries of behaviour-specific time allocation during the afternoons as a function of species, blackglobe temperature, hour, month and individual.

a) browsing

| Component | Term | Estimate | Std Error | t-value | p-value |  |
| --- | --- | --- | --- | --- | --- | --- |
| A. parametric coefficients | (Intercept) | 0.134 | 0.017 | 7.935 | 0.0000 | *** |
| kudu | 0.205 | 0.023 | 8.739 | 0.0000 | *** |
| eland | 0.074 | 0.023 | 3.155 | 0.0016 | ** |
| Component | Term | edf | Ref. df | F-value | p-value |  |
| B. smooth terms | s(blackglobe):springbok | 0.930 | 9.000 | 1.677 | 0.0001 | *** |
| s(blackglobe):kudu | 5.660 | 9.000 | 5.627 | 0.0000 | *** |
| s(blackglobe):eland | 0.888 | 9.000 | 1.435 | 0.0027 | ** |
| s(hour):springbok | 2.313 | 5.000 | 3.464 | 0.0001 | *** |
| s(hour):kudu | 3.217 | 5.000 | 15.013 | 0.0000 | *** |
| s(hour):eland | 3.703 | 5.000 | 18.821 | 0.0000 | *** |
| s(month):springbok | 1.822 | 2.000 | 28.054 | 0.0000 | *** |
| s(month):kudu | 1.669 | 2.000 | 250.224 | 0.0000 | *** |
| s(month):eland | 1.901 | 2.000 | 20.838 | 0.0000 | *** |
| s(id) | 19.815 | 21.000 | 29.107 | 0.0000 | *** |
| Signif. codes: 0 <= '***' < 0.001 < '**' < 0.01 < '*' < 0.05 |
| Adjusted R-squared: 0.650, Deviance explained 0.656 |
| fREML : -2717.748, Scale est: 0.00622, N: 2502 |

b) grazing

| Component | Term | Estimate | Std Error | t-value | p-value |  |
| --- | --- | --- | --- | --- | --- | --- |
| A. parametric coefficients | (Intercept) | 0.281 | 0.018 | 15.406 | 0.0000 | *** |
| kudu | -0.103 | 0.025 | -4.092 | 0.0000 | *** |
| eland | -0.068 | 0.025 | -2.686 | 0.0073 | ** |
| Component | Term | edf | Ref. df | F-value | p-value |  |
| B. smooth terms | s(blackglobe):springbok | 4.985 | 9.000 | 44.146 | 0.0000 | *** |
| s(blackglobe):kudu | 2.139 | 9.000 | 2.718 | 0.0001 | *** |
| s(blackglobe):eland | 4.673 | 9.000 | 33.730 | 0.0000 | *** |
| s(hour):springbok | 4.517 | 5.000 | 16.640 | 0.0000 | *** |
| s(hour):kudu | 1.829 | 5.000 | 1.263 | 0.0279 | * |
| s(hour):eland | 4.576 | 5.000 | 67.784 | 0.0000 | *** |
| s(month):springbok | 1.986 | 2.000 | 161.583 | 0.0000 | *** |
| s(month):kudu | 1.951 | 2.000 | 103.437 | 0.0000 | *** |
| s(month):eland | 1.595 | 2.000 | 4.570 | 0.0042 | ** |
| s(id) | 19.824 | 21.000 | 23.902 | 0.0000 | *** |
| Signif. codes: 0 <= '***' < 0.001 < '**' < 0.01 < '*' < 0.05 |
| Adjusted R-squared: 0.616, Deviance explained 0.623 |
| fREML : -2535.752, Scale est: 0.00713, N: 2502 |

c) walking

| Component | Term | Estimate | Std Error | t-value | p-value |  |
| --- | --- | --- | --- | --- | --- | --- |
| A. parametric coefficients | (Intercept) | 0.214 | 0.011 | 19.071 | 0.0000 | *** |
| kudu | -0.133 | 0.016 | -8.588 | 0.0000 | *** |
| eland | -0.135 | 0.016 | -8.718 | 0.0000 | *** |
| Component | Term | edf | Ref. df | F-value | p-value |  |
| B. smooth terms | s(blackglobe):springbok | 2.255 | 9.000 | 1.054 | 0.0068 | ** |
| s(blackglobe):kudu | 2.293 | 9.000 | 3.641 | 0.0000 | *** |
| s(blackglobe):eland | 0.811 | 9.000 | 0.192 | 0.1323 |  |
| s(hour):springbok | 3.651 | 5.000 | 13.437 | 0.0000 | *** |
| s(hour):kudu | 2.327 | 5.000 | 8.844 | 0.0000 | *** |
| s(hour):eland | 2.619 | 5.000 | 10.797 | 0.0000 | *** |
| s(month):springbok | 1.871 | 2.000 | 32.304 | 0.0000 | *** |
| s(month):kudu | 1.511 | 2.000 | 30.877 | 0.0000 | *** |
| s(month):eland | 0.000 | 2.000 | 0.000 | 0.3713 |  |
| s(id) | 19.523 | 21.000 | 21.520 | 0.0000 | *** |
| Signif. codes: 0 <= '***' < 0.001 < '**' < 0.01 < '*' < 0.05 |
| Adjusted R-squared: 0.613, Deviance explained 0.619 |
| fREML : -3439.893, Scale est: 0.00354, N: 2502 |

d) ruminating

| Component | Term | Estimate | Std Error | t-value | p-value |  |
| --- | --- | --- | --- | --- | --- | --- |
| A. parametric coefficients | (Intercept) | 0.215 | 0.018 | 12.266 | 0.0000 | *** |
| kudu | -0.063 | 0.024 | -2.598 | 0.0094 | ** |
| eland | -0.048 | 0.024 | -1.959 | 0.0503 | . |
| Component | Term | edf | Ref. df | F-value | p-value |  |
| B. smooth terms | s(blackglobe):springbok | 3.063 | 9.000 | 20.945 | 0.0000 | *** |
| s(blackglobe):kudu | 2.195 | 9.000 | 2.780 | 0.0005 | *** |
| s(blackglobe):eland | 2.441 | 9.000 | 2.507 | 0.0003 | *** |
| s(hour):springbok | 4.443 | 5.000 | 23.273 | 0.0000 | *** |
| s(hour):kudu | 3.483 | 5.000 | 12.877 | 0.0000 | *** |
| s(hour):eland | 4.518 | 5.000 | 52.638 | 0.0000 | *** |
| s(month):springbok | 1.964 | 2.000 | 97.703 | 0.0000 | *** |
| s(month):kudu | 1.296 | 2.000 | 13.316 | 0.0000 | *** |
| s(month):eland | 1.766 | 2.000 | 35.882 | 0.0000 | *** |
| s(id) | 19.864 | 21.000 | 30.448 | 0.0000 | *** |
| Signif. codes: 0 <= '***' < 0.001 < '**' < 0.01 < '*' < 0.05 |
| Adjusted R-squared: 0.509, Deviance explained 0.518 |
| fREML : -2681.247, Scale est: 0.00638, N: 2502 |

e) resting

| Component | Term | Estimate | Std Error | t-value | p-value |  |
| --- | --- | --- | --- | --- | --- | --- |
| A. parametric coefficients | (Intercept) | 0.135 | 0.025 | 5.340 | 0.0000 | *** |
| kudu | 0.089 | 0.035 | 2.505 | 0.0123 | * |
| eland | 0.155 | 0.035 | 4.382 | 0.0000 | *** |
| Component | Term | edf | Ref. df | F-value | p-value |  |
| B. smooth terms | s(blackglobe):springbok | 0.902 | 9.000 | 1.282 | 0.0012 | ** |
| s(blackglobe):kudu | 0.976 | 9.000 | 13.505 | 0.0000 | *** |
| s(blackglobe):eland | 3.542 | 9.000 | 9.214 | 0.0000 | *** |
| s(hour):springbok | 3.301 | 5.000 | 8.492 | 0.0000 | *** |
| s(hour):kudu | 2.763 | 5.000 | 5.333 | 0.0001 | *** |
| s(hour):eland | 4.034 | 5.000 | 28.827 | 0.0000 | *** |
| s(month):springbok | 0.910 | 2.000 | 8.031 | 0.0009 | *** |
| s(month):kudu | 1.885 | 2.000 | 21.760 | 0.0000 | *** |
| s(month):eland | 1.201 | 2.000 | 36.082 | 0.0000 | *** |
| s(id) | 20.322 | 21.000 | 63.696 | 0.0000 | *** |
| Signif. codes: 0 <= '***' < 0.001 < '**' < 0.01 < '*' < 0.05 |
| Adjusted R-squared: 0.602, Deviance explained 0.608 |
| fREML : -2466.350, Scale est: 0.00761, N: 2502 |

## Analysis of afternoon behaviour-specific energy expenditure

Table S3: Generalised Additive Model summaries of behaviour-specific mean DBA (Dynamic Body Acceleration, a proxy for energy expenditure per unit time) during the afternoons as a function of species, blackglobe temperature, hour, month and individual.

a) browsing

| Component | Term | Estimate | Std Error | t-value | p-value |  |
| --- | --- | --- | --- | --- | --- | --- |
| A. parametric coefficients | (Intercept) | 2.014 | 0.057 | 35.162 | 0.0000 | *** |
| kudu | -1.127 | 0.085 | -13.313 | 0.0000 | *** |
| eland | -1.001 | 0.085 | -11.813 | 0.0000 | *** |
| Component | Term | edf | Ref. df | F-value | p-value |  |
| B. smooth terms | s(temp):springbok | 2.849 | 4.000 | 16.866 | 0.0000 | *** |
| s(temp):kudu | 0.427 | 4.000 | 0.189 | 0.1808 |  |
| s(temp):eland | 2.198 | 4.000 | 18.935 | 0.0000 | *** |
| s(hour):springbok | 2.393 | 4.000 | 5.708 | 0.0000 | *** |
| s(hour):kudu | 1.814 | 4.000 | 2.730 | 0.0017 | ** |
| s(hour):eland | 3.359 | 4.000 | 22.732 | 0.0000 | *** |
| s(month):springbok | 1.935 | 2.000 | 89.060 | 0.0000 | *** |
| s(month):kudu | 0.803 | 2.000 | 2.088 | 0.0237 | * |
| s(month):eland | 0.965 | 2.000 | 16.158 | 0.0000 | *** |
| s(id) | 22.000 | 23.000 | 30.927 | 0.0000 | *** |
| Signif. codes: 0 <= '***' < 0.001 < '**' < 0.01 < '*' < 0.05 |
| Adjusted R-squared: 0.534, Deviance explained 0.537 |
| fREML : 5254.744, Scale est: 0.241, N: 7317 |

b) grazing

| Component | Term | Estimate | Std Error | t-value | p-value |  |
| --- | --- | --- | --- | --- | --- | --- |
| A. parametric coefficients | (Intercept) | 2.998 | 0.075 | 40.174 | 0.0000 | *** |
| kudu | -1.734 | 0.111 | -15.586 | 0.0000 | *** |
| eland | -1.412 | 0.111 | -12.699 | 0.0000 | *** |
| Component | Term | edf | Ref. df | F-value | p-value |  |
| B. smooth terms | s(temp):springbok | 2.506 | 4.000 | 8.859 | 0.0000 | *** |
| s(temp):kudu | 1.425 | 4.000 | 1.624 | 0.0111 | * |
| s(temp):eland | 0.900 | 4.000 | 2.451 | 0.0014 | ** |
| s(hour):springbok | 0.432 | 4.000 | 0.200 | 0.1842 |  |
| s(hour):kudu | 2.161 | 4.000 | 6.571 | 0.0000 | *** |
| s(hour):eland | 1.870 | 4.000 | 2.797 | 0.0022 | ** |
| s(month):springbok | 1.977 | 2.000 | 1,306.159 | 0.0000 | *** |
| s(month):kudu | 1.925 | 2.000 | 41.237 | 0.0000 | *** |
| s(month):eland | 1.714 | 2.000 | 24.462 | 0.0000 | *** |
| s(id) | 22.284 | 23.000 | 42.231 | 0.0000 | *** |
| Signif. codes: 0 <= '***' < 0.001 < '**' < 0.01 < '*' < 0.05 |
| Adjusted R-squared: 0.682, Deviance explained 0.684 |
| fREML : 6647.215, Scale est: 0.354, N: 7304 |

c) walking

| Component | Term | Estimate | Std Error | t-value | p-value |  |
| --- | --- | --- | --- | --- | --- | --- |
| A. parametric coefficients | (Intercept) | 2.994 | 0.120 | 24.951 | 0.0000 | *** |
| kudu | -0.679 | 0.178 | -3.808 | 0.0001 | *** |
| eland | -0.457 | 0.178 | -2.566 | 0.0103 | * |
| Component | Term | edf | Ref. df | F-value | p-value |  |
| B. smooth terms | s(temp):springbok | 0.000 | 4.000 | 0.000 | 0.4841 |  |
| s(temp):kudu | 1.386 | 4.000 | 3.745 | 0.0001 | *** |
| s(temp):eland | 1.305 | 4.000 | 8.156 | 0.0000 | *** |
| s(hour):springbok | 0.000 | 4.000 | 0.000 | 0.9660 |  |
| s(hour):kudu | 1.790 | 4.000 | 3.559 | 0.0005 | *** |
| s(hour):eland | 3.459 | 4.000 | 17.406 | 0.0000 | *** |
| s(month):springbok | 1.488 | 2.000 | 30.727 | 0.0000 | *** |
| s(month):kudu | 1.121 | 2.000 | 4.727 | 0.0024 | ** |
| s(month):eland | 0.185 | 2.000 | 0.121 | 0.2673 |  |
| s(id) | 21.991 | 23.000 | 32.510 | 0.0000 | *** |
| Signif. codes: 0 <= '***' < 0.001 < '**' < 0.01 < '*' < 0.05 |
| Adjusted R-squared: 0.196, Deviance explained 0.201 |
| fREML : 8255.254, Scale est: 0.894, N: 6011 |

d) ruminating

| Component | Term | Estimate | Std Error | t-value | p-value |  |
| --- | --- | --- | --- | --- | --- | --- |
| A. parametric coefficients | (Intercept) | 0.466 | 0.013 | 35.618 | 0.0000 | *** |
| kudu | -0.162 | 0.019 | -8.310 | 0.0000 | *** |
| eland | -0.066 | 0.019 | -3.391 | 0.0007 | *** |
| Component | Term | edf | Ref. df | F-value | p-value |  |
| B. smooth terms | s(temp):springbok | 3.729 | 4.000 | 11.349 | 0.0000 | *** |
| s(temp):kudu | 0.000 | 4.000 | 0.000 | 0.4123 |  |
| s(temp):eland | 0.870 | 4.000 | 1.920 | 0.0053 | ** |
| s(hour):springbok | 1.888 | 4.000 | 3.199 | 0.0009 | *** |
| s(hour):kudu | 2.194 | 4.000 | 2.292 | 0.0107 | * |
| s(hour):eland | 2.201 | 4.000 | 13.149 | 0.0000 | *** |
| s(month):springbok | 0.960 | 2.000 | 20.921 | 0.0000 | *** |
| s(month):kudu | 1.628 | 2.000 | 5.596 | 0.0015 | ** |
| s(month):eland | 0.971 | 2.000 | 20.367 | 0.0000 | *** |
| s(id) | 21.980 | 23.000 | 31.956 | 0.0000 | *** |
| Signif. codes: 0 <= '***' < 0.001 < '**' < 0.01 < '*' < 0.05 |
| Adjusted R-squared: 0.325, Deviance explained 0.329 |
| fREML : -4755.678, Scale est: 0.013, N: 6423 |

e) resting

| Component | Term | Estimate | Std Error | t-value | p-value |  |
| --- | --- | --- | --- | --- | --- | --- |
| A. parametric coefficients | (Intercept) | 0.253 | 0.013 | 19.223 | 0.0000 | *** |
| kudu | -0.120 | 0.019 | -6.424 | 0.0000 | *** |
| eland | 0.007 | 0.019 | 0.353 | 0.7237 |  |
| Component | Term | edf | Ref. df | F-value | p-value |  |
| B. smooth terms | s(temp):springbok | 0.913 | 4.000 | 2.824 | 0.0007 | *** |
| s(temp):kudu | 1.594 | 4.000 | 2.334 | 0.0025 | ** |
| s(temp):eland | 0.775 | 4.000 | 0.893 | 0.0334 | * |
| s(hour):springbok | 0.000 | 4.000 | 0.000 | 0.5381 |  |
| s(hour):kudu | 1.442 | 4.000 | 1.495 | 0.0145 | * |
| s(hour):eland | 1.782 | 4.000 | 7.158 | 0.0000 | *** |
| s(month):springbok | 0.000 | 2.000 | 0.000 | 0.7436 |  |
| s(month):kudu | 0.965 | 2.000 | 7.573 | 0.0001 | *** |
| s(month):eland | 0.958 | 2.000 | 11.572 | 0.0000 | *** |
| s(id) | 17.948 | 23.000 | 4.933 | 0.0000 | *** |
| Signif. codes: 0 <= '***' < 0.001 < '**' < 0.01 < '*' < 0.05 |
| Adjusted R-squared: 0.0812, Deviance explained 0.0851 |
| fREML : 93.997, Scale est: 0.0595, N: 6611 |

# Comparison of behaviour-specific time allocation between hot days and cool days

Table S4: Generalised Additive Model summaries of diel patterns of behaviour-specific time allocation as a function of hot / cool day, hour of the day and individual.

a) browsing

| Springbok |  |  |  |  |  |  |
| --- | --- | --- | --- | --- | --- | --- |
| Component | Term | Estimate | Std Error | t-value | p-value |  |
| A. parametric coefficients | (Intercept) | 0.105 | 0.007 | 14.488 | 0.0000 | *** |
| hot | -0.012 | 0.004 | -2.707 | 0.0068 | ** |
| Component | Term | edf | Ref. df | F-value | p-value |  |
| B. smooth terms | s(hour) | 9.729 | 19.000 | 5.449 | 0.0000 | *** |
| s(hour):cool | 0.001 | 19.000 | 0.000 | 0.0044 | ** |
| s(hour):hot | 2.016 | 19.000 | 0.507 | 0.0043 | ** |
| s(id) | 5.932 | 7.000 | 5.796 | 0.0000 | *** |
| Adjusted R-squared: 0.105, Deviance explained 0.111 |
| fREML : -1984.854, Scale est: 0.0113, N: 2454 |
| Kudu |  |  |  |  |  |  |
| Component | Term | Estimate | Std Error | t-value | p-value |  |
| A. parametric coefficients | (Intercept) | 0.339 | 0.012 | 27.472 | 0.0000 | *** |
| hot | -0.019 | 0.007 | -2.667 | 0.0077 | ** |
| Component | Term | edf | Ref. df | F-value | p-value |  |
| B. smooth terms | s(hour) | 14.802 | 19.000 | 18.485 | 0.0000 | *** |
| s(hour):cool | 0.002 | 19.000 | 0.000 | 0.0251 | * |
| s(hour):hot | 1.568 | 19.000 | 0.293 | 0.0236 | * |
| s(id) | 6.346 | 7.000 | 9.769 | 0.0000 | *** |
| Adjusted R-squared: 0.107, Deviance explained 0.113 |
| fREML : -323.466, Scale est: 0.0479, N: 3702 |
| Eland |  |  |  |  |  |  |
| Component | Term | Estimate | Std Error | t-value | p-value |  |
| A. parametric coefficients | (Intercept) | 0.190 | 0.018 | 10.371 | 0.0000 | *** |
| hot | 0.003 | 0.005 | 0.620 | 0.5355 |  |
| Component | Term | edf | Ref. df | F-value | p-value |  |
| B. smooth terms | s(hour) | 3.679 | 19.000 | 0.737 | 0.0016 | ** |
| s(hour):cool | 0.000 | 19.000 | 0.000 | 0.1624 |  |
| s(hour):hot | 1.634 | 19.000 | 0.286 | 0.0259 | * |
| s(id) | 6.865 | 7.000 | 50.619 | 0.0000 | *** |
| Adjusted R-squared: 0.0965, Deviance explained 0.0998 |
| fREML : -1664.504, Scale est: 0.0233, N: 3682 |
| Signif. codes: 0 <= '***' < 0.001 < '**' < 0.01 < '*' < 0.05 |

b) grazing

| Springbok |  |  |  |  |  |  |
| --- | --- | --- | --- | --- | --- | --- |
| Component | Term | Estimate | Std Error | t-value | p-value |  |
| A. parametric coefficients | (Intercept) | 0.266 | 0.014 | 18.918 | 0.0000 | *** |
| hot | -0.001 | 0.009 | -0.073 | 0.9420 |  |
| Component | Term | edf | Ref. df | F-value | p-value |  |
| B. smooth terms | s(hour) | 16.675 | 19.000 | 35.671 | 0.0000 | *** |
| s(hour):cool | 0.000 | 19.000 | 0.000 | 0.0107 | * |
| s(hour):hot | 5.505 | 19.000 | 2.626 | 0.0000 | *** |
| s(id) | 5.801 | 7.000 | 5.095 | 0.0000 | *** |
| Adjusted R-squared: 0.313, Deviance explained 0.321 |
| fREML : -180.120, Scale est: 0.048, N: 2454 |
| Kudu |  |  |  |  |  |  |
| Component | Term | Estimate | Std Error | t-value | p-value |  |
| A. parametric coefficients | (Intercept) | 0.177 | 0.010 | 17.446 | 0.0000 | *** |
| hot | -0.013 | 0.005 | -2.509 | 0.0122 | * |
| Component | Term | edf | Ref. df | F-value | p-value |  |
| B. smooth terms | s(hour) | 15.846 | 19.000 | 31.117 | 0.0000 | *** |
| s(hour):cool | 0.000 | 19.000 | 0.000 | 0.4230 |  |
| s(hour):hot | 0.000 | 19.000 | 0.000 | 0.4221 |  |
| s(id) | 6.524 | 7.000 | 13.581 | 0.0000 | *** |
| Adjusted R-squared: 0.157, Deviance explained 0.163 |
| fREML : -1575.912, Scale est: 0.0243, N: 3702 |
| Eland |  |  |  |  |  |  |
| Component | Term | Estimate | Std Error | t-value | p-value |  |
| A. parametric coefficients | (Intercept) | 0.275 | 0.011 | 25.931 | 0.0000 | *** |
| hot | -0.022 | 0.007 | -3.225 | 0.0013 | ** |
| Component | Term | edf | Ref. df | F-value | p-value |  |
| B. smooth terms | s(hour) | 17.058 | 19.000 | 46.583 | 0.0000 | *** |
| s(hour):cool | 0.001 | 19.000 | 0.000 | 0.0017 | ** |
| s(hour):hot | 3.762 | 19.000 | 2.794 | 0.0000 | *** |
| s(id) | 6.148 | 7.000 | 7.264 | 0.0000 | *** |
| Adjusted R-squared: 0.238, Deviance explained 0.244 |
| fREML : -447.136, Scale est: 0.0444, N: 3682 |
| Signif. codes: 0 <= '***' < 0.001 < '**' < 0.01 < '*' < 0.05 |

c) walking

| Springbok |  |  |  |  |  |  |
| --- | --- | --- | --- | --- | --- | --- |
| Component | Term | Estimate | Std Error | t-value | p-value |  |
| A. parametric coefficients | (Intercept) | 0.174 | 0.012 | 14.764 | 0.0000 | *** |
| hot | -0.007 | 0.006 | -1.129 | 0.2591 |  |
| Component | Term | edf | Ref. df | F-value | p-value |  |
| B. smooth terms | s(hour) | 14.687 | 19.000 | 22.246 | 0.0000 | *** |
| s(hour):cool | 0.154 | 19.000 | 0.010 | 0.0889 | . |
| s(hour):hot | 5.661 | 19.000 | 1.702 | 0.0000 | *** |
| s(id) | 6.153 | 7.000 | 8.394 | 0.0000 | *** |
| Adjusted R-squared: 0.256, Deviance explained 0.264 |
| fREML : -1038.420, Scale est: 0.024, N: 2454 |
| Kudu |  |  |  |  |  |  |
| Component | Term | Estimate | Std Error | t-value | p-value |  |
| A. parametric coefficients | (Intercept) | 0.075 | 0.006 | 11.689 | 0.0000 | *** |
| hot | 0.005 | 0.003 | 1.542 | 0.1231 |  |
| Component | Term | edf | Ref. df | F-value | p-value |  |
| B. smooth terms | s(hour) | 14.304 | 19.000 | 26.441 | 0.0000 | *** |
| s(hour):cool | 0.000 | 19.000 | 0.000 | 0.8940 |  |
| s(hour):hot | 0.000 | 19.000 | 0.000 | 0.8807 |  |
| s(id) | 6.450 | 7.000 | 11.664 | 0.0000 | *** |
| Adjusted R-squared: 0.135, Deviance explained 0.140 |
| fREML : -3047.083, Scale est: 0.011, N: 3702 |
| Eland |  |  |  |  |  |  |
| Component | Term | Estimate | Std Error | t-value | p-value |  |
| A. parametric coefficients | (Intercept) | 0.101 | 0.007 | 15.279 | 0.0000 | *** |
| hot | 0.025 | 0.005 | 5.236 | 0.0000 | *** |
| Component | Term | edf | Ref. df | F-value | p-value |  |
| B. smooth terms | s(hour) | 14.064 | 19.000 | 14.642 | 0.0000 | *** |
| s(hour):cool | 0.000 | 19.000 | 0.000 | 0.0021 | ** |
| s(hour):hot | 3.887 | 19.000 | 1.506 | 0.0000 | *** |
| s(id) | 5.915 | 7.000 | 5.553 | -0.0000 | *** |
| Adjusted R-squared: 0.111, Deviance explained 0.117 |
| fREML : -1823.321, Scale est: 0.0212, N: 3682 |
| Signif. codes: 0 <= '***' < 0.001 < '**' < 0.01 < '*' < 0.05 |

d) ruminating

| Springbok |  |  |  |  |  |  |
| --- | --- | --- | --- | --- | --- | --- |
| Component | Term | Estimate | Std Error | t-value | p-value |  |
| A. parametric coefficients | (Intercept) | 0.157 | 0.022 | 7.003 | 0.0000 | *** |
| hot | 0.031 | 0.007 | 4.416 | 0.0000 | *** |
| Component | Term | edf | Ref. df | F-value | p-value |  |
| B. smooth terms | s(hour) | 16.894 | 19.000 | 20.751 | 0.0000 | *** |
| s(hour):cool | 0.522 | 19.000 | 0.057 | 0.0290 | * |
| s(hour):hot | 7.977 | 19.000 | 7.000 | 0.0000 | *** |
| s(id) | 6.714 | 7.000 | 40.742 | 0.0000 | *** |
| Adjusted R-squared: 0.332, Deviance explained 0.341 |
| fREML : -777.179, Scale est: 0.0292, N: 2454 |
| Kudu |  |  |  |  |  |  |
| Component | Term | Estimate | Std Error | t-value | p-value |  |
| A. parametric coefficients | (Intercept) | 0.167 | 0.014 | 11.632 | 0.0000 | *** |
| hot | 0.002 | 0.006 | 0.298 | 0.7658 |  |
| Component | Term | edf | Ref. df | F-value | p-value |  |
| B. smooth terms | s(hour) | 16.333 | 19.000 | 48.112 | 0.0000 | *** |
| s(hour):cool | 0.000 | 19.000 | 0.000 | 0.7101 |  |
| s(hour):hot | 0.000 | 19.000 | 0.000 | 0.6834 |  |
| s(id) | 6.702 | 7.000 | 22.815 | 0.0000 | *** |
| Adjusted R-squared: 0.225, Deviance explained 0.230 |
| fREML : -1117.059, Scale est: 0.0311, N: 3702 |
| Eland |  |  |  |  |  |  |
| Component | Term | Estimate | Std Error | t-value | p-value |  |
| A. parametric coefficients | (Intercept) | 0.176 | 0.014 | 12.609 | 0.0000 | *** |
| hot | -0.030 | 0.006 | -4.932 | 0.0000 | *** |
| Component | Term | edf | Ref. df | F-value | p-value |  |
| B. smooth terms | s(hour) | 16.443 | 19.000 | 29.425 | 0.0000 | *** |
| s(hour):cool | 2.981 | 19.000 | 1.035 | 0.0001 | *** |
| s(hour):hot | 0.000 | 19.000 | 0.000 | 0.0053 | ** |
| s(id) | 6.647 | 7.000 | 18.975 | 0.0000 | *** |
| Adjusted R-squared: 0.191, Deviance explained 0.197 |
| fREML : -940.398, Scale est: 0.034, N: 3682 |
| Signif. codes: 0 <= '***' < 0.001 < '**' < 0.01 < '*' < 0.05 |

e) resting

| Springbok |  |  |  |  |  |  |
| --- | --- | --- | --- | --- | --- | --- |
| Component | Term | Estimate | Std Error | t-value | p-value |  |
| A. parametric coefficients | (Intercept) | 0.272 | 0.030 | 9.233 | 0.0000 | *** |
| hot | -0.004 | 0.009 | -0.486 | 0.6271 |  |
| Component | Term | edf | Ref. df | F-value | p-value |  |
| B. smooth terms | s(hour) | 16.350 | 19.000 | 53.838 | 0.0000 | *** |
| s(hour):cool | 0.000 | 19.000 | 0.000 | 0.0008 | *** |
| s(hour):hot | 4.241 | 19.000 | 0.691 | 0.0067 | ** |
| s(id) | 6.728 | 7.000 | 45.100 | 0.0000 | *** |
| Adjusted R-squared: 0.462, Deviance explained 0.469 |
| fREML : -177.567, Scale est: 0.048, N: 2454 |
| Kudu |  |  |  |  |  |  |
| Component | Term | Estimate | Std Error | t-value | p-value |  |
| A. parametric coefficients | (Intercept) | 0.214 | 0.019 | 11.472 | 0.0000 | *** |
| hot | 0.024 | 0.007 | 3.454 | 0.0006 | *** |
| Component | Term | edf | Ref. df | F-value | p-value |  |
| B. smooth terms | s(hour) | 16.416 | 19.000 | 33.166 | 0.0000 | *** |
| s(hour):cool | 0.001 | 19.000 | 0.000 | 0.0072 | ** |
| s(hour):hot | 1.901 | 19.000 | 0.420 | 0.0095 | ** |
| s(id) | 6.743 | 7.000 | 26.075 | 0.0000 | *** |
| Adjusted R-squared: 0.195, Deviance explained 0.200 |
| fREML : -402.511, Scale est: 0.0456, N: 3702 |
| Eland |  |  |  |  |  |  |
| Component | Term | Estimate | Std Error | t-value | p-value |  |
| A. parametric coefficients | (Intercept) | 0.224 | 0.021 | 10.620 | 0.0000 | *** |
| hot | 0.018 | 0.006 | 2.885 | 0.0039 | ** |
| Component | Term | edf | Ref. df | F-value | p-value |  |
| B. smooth terms | s(hour) | 15.724 | 19.000 | 24.989 | 0.0000 | *** |
| s(hour):cool | 0.000 | 19.000 | 0.000 | 0.0011 | ** |
| s(hour):hot | 4.504 | 19.000 | 3.239 | 0.0000 | *** |
| s(id) | 6.840 | 7.000 | 42.562 | 0.0000 | *** |
| Adjusted R-squared: 0.227, Deviance explained 0.233 |
| fREML : -827.341, Scale est: 0.0361, N: 3682 |
| Signif. codes: 0 <= '***' < 0.001 < '**' < 0.01 < '*' < 0.05 |
